# Microenvironment Modulates Osteogenic Cell Lineage Commitment in Differentiated Embryonic Stem Cells

**DOI:** 10.1371/journal.pone.0009663

**Published:** 2010-03-12

**Authors:** Akihiro Yamashita, Sandi Nishikawa, Derrick E. Rancourt

**Affiliations:** Department of Biochemistry and Molecular Biology, University of Calgary, Calgary, Alberta, Canada; Health Canada, Canada

## Abstract

**Background:**

Due to their self-renewal, embryonic stem cells (ESCs) are attractive cells for applications in regenerative medicine and tissue engineering. Although ESC differentiation has been used as a platform for generating bone *in vitro* and *in vivo*, the results have been unsatisfactory at best. It is possible that the traditional culture methods, which have been used, are not optimal and that other approaches must be explored.

**Methodology/Principal Findings:**

ESCs were differentiated into osteoblast lineage using a micro-mass approach. In response to osteogenic differentiation medium, many cells underwent apoptosis, while others left the micro-mass, forming small aggregates in suspension. These aggregates were cultured in three different culture conditions (adhesion, static suspension, and stirred suspension), then examined for osteogenic potential *in vitro* and *in vivo*. In adhesion culture, ESCs primed to become osteoblasts recommitted to the adipocyte lineage *in vitro*. In a static suspension culture, resulting porous aggregates expressed osteoblasts markers and formed bone *in vivo* via intermembranous ossification. In a stirred suspension culture, resulting non-porous aggregates suppressed osteoblast differentiation in favor of expanding progenitor cells.

**Conclusions/Significance:**

We demonstrate that microenvironment modulates cell fate and subsequent tissue formation during ESC differentiation. For effective tissue engineering using ESCs, it is important to develop optimized cell culture/differentiation conditions based upon the influence of microenvironment.

## Introduction

Embryonic stem cells (ESCs) provide an unlimited supply of progenitor cells for tissue engineering [1], [2]. However, successful use of ESCs for clinical application requires the development of efficient methods to differentiate cells into specific lineages *in vitro* and reconstituting tissue function *in vivo*. Using traditional culture methods, the osteogenic differentiation of ESCs has been established *in vitro* [3]–[7]. Although differentiated ESCs express osteogenic makers and calcify *in vitro*, previous studies suggest that it is difficult to form functional bone *in vivo* [8]. We hypothesized that there may be factors disturbing ESC osteoblast lineage commitment and bone formation.

Osteoblasts, adipocytes and chondrocytes are believed to share a common mesenchymal progenitor and these cells show the differentiation plasticity, resulting in the possible reciprocal relationship of these lineages [9], [10]. In particular, osteogenesis is dominant over adipogenesis in the bone marrow derived MSCs of younger adults, while this balance is usually reversed in the bone marrow of older adults including patients with osteoporosis [11], [12]. Such fate decisions are regulated in part by transcription factors [12]. For example, we have previously reported that mesenchymal differentiation can be switched from the adipocyte to the osteoblast lineage simply by suppressing PPARγ transactivation in cells [13].

In addition to transcription factors, microenvironment may also regulate cell fate and subsequent tissue formation [14]. By combining parameters such as cell density and the organization of multi-cellular structures in three dimensional (3D) space, cell fate is likely determined through the compliance of local environment and force equilibration through cell-cell/cell-matrix contacts. For example, 3D culture systems, which force cell condensation in the form of a pellet or micro-mass is used to induce the chondrogenic differentiation of MSCs [15].

In this study, we developed a novel method to differentiate ESCs towards the osteoblast lineage and to form bone *in vivo* using aggregates derived from micro-mass culture. We show that a microenvironment appears to significantly regulate osteoblast-adipocyte and osteoblast-precursor cell balances during osteogenic differentiation.

## Materials and Methods

### Mouse ESC culture

The mouse D3 ESC line was cultured on mitomycin C-treated (10 µg/ml, 37°C for 2 hours; Sigma) mouse embryonic fibroblast (MEF) feeder layers on gelatin-coated tissue culture dishes. Medium consisted of high glucose DMEM (4.5 g Glucose/l, Gibco) containing 15% FBS (Gibco), 1% non-essential amino acids (NEAA, Invitrogen), 50 U/ml penicillin and 50 µg/ml streptomycin (Pen/Strep Invitrogen), 0.1 mM 2-mercaptoethanol (βME, Invitrogen) and 1000 U/ml leukemia inhibitory factor (LIF, Invitrogen), as described previously [16].

### ESC micro-mass culture

Micro-mass culture was performed as described previously [16]. Briefly, ESCs were dissociated using 0.1% trypsin-EDTA. The dissociated cells were cultured at a high-density 1.0×10^5^ cells per 10 µl spot for 2 hours. After incubation, medium was gently added to each dish as to not dissociate aggregates. For osteoblast differentiation, we used six media types containing the same base: DMEM, 1% NEAA, Pen/Strep, βME. To the base medium the following factors were added (i) FBS-basic: 15% FBS (Invitrogen), (ii) FBS-Dex: 15% FBS, 50 µg/ml Ascorbic acid (AA) (Sigma), 10 mM β-glycerophosphate (β-GP) (Sigma), 100 nM dexamethasone (Dex) (Sigma), (iii) FBS-VD3: 15% FBS, 50 µg/ml AA, 10 mM β-GP, 50 nM 1,25-OH_2_ Vitamin D_3_ (VD3) (Calbiochem). (iv) KSR-basic: 15% Knockout Serum Replacement (KSR) (Invitrogen), (v) KSR-Dex: 15% KSR, 50 µg/ml AA, 10 mM β-GP, 100 nM Dex (vi) KSR-VD3: 15% KSR, 50 µg/ml AA, 10 mM β-GP, 50 nM VD3.

### Adherent, static suspension and stirred suspension culture

Aggregates were formed spontaneously within micro-drops. At day 5, these aggregates were expanded via adhesion or suspension culture. For adhesion culture, we used gelatin-coated tissue culture dishes and examined six media types, FBS-basic, FBS-Dex, FBS-VD3, KSR-basic, KSR-Dex and KSR-VD3. In static suspension culture, we used a 6.0 cm petri dish and examined three media, KSR-basic, KSR-Dex and KSR-VD3. For stirred suspension, the culture was agitated at 100 rpm using KSR-VD3. Following 30 days differentiation in stirred suspension some aggregates were transferred to culture dishes. After 2 days, resulting aggregate outgrowths were dissociated and cultured again in new dishes.

### RNA isolation and real-time RT-PCR

Total RNA was isolated from cell culture using the RNeasy Mini Kit with on-column DNase I digestion (Qiagen) according to the manufacturer's instructions. The amount of total RNA was measured using a Bio Photometer. Two micrograms of total RNA were used as a template for cDNA synthesis using the Super Script III First-Strand Synthesys System (Invitrogen). The PCR reactions (20 µl) were carried out using Taq DNA Polymerase (Invitrogen) according to the manufacturer's instructions. PCR conditions were: 3 min at 94°C, 30 sec denaturation at 94°C, 45 sec annealing at 55°C, and 1 min 30 sec extension at 72°C. Primers were designed based on the mouse sequence and BLASTed for their specificity at the National Center for Biotechnology Information (NCBI). Primer sequences are described in Table 1.

**Table 1:** Primer sequences.

Gene	Primer Sequence (5′-3′)
Cbfa1	F: CCGCACGACAACCGCACCAT
	R: CGCTCCGGCCCACAAATCTC
OCN	F: TCTCTCTGCTCACTCTGCTGG
	R: ACCGTAGATGCGTTTGTAGGC
PPARγ:	F: AAGAGCTGACCCAATGGTTG
	R: TCCATAGTGGAAGCCTGATGC
aP2	F: ATGTGTGATGCCTTTGTGGGA
	R: TGCCCTTTCATAAACTCTTGT
β-actin	F: GGCCCAGAGCAAGAGAGGTATCC
	R: ACGCACGATTTCCCTCTCAGC

Amplified products were used to derive standard curves for quantitative RT-PCR (real-time RT-PCR). Quantitative PCR was performed in an iCycler iQ system using a SYBR green PCR master mix (Biorad, Hercules, CA), with the following cycle conditions: an initial denaturation step at activation of enzyme at 95°C for 3 min, 45 cycles of denaturation at 95°C for 30 sec, annealing at 57°C for 30 sec and extension at 72°C for 30 sec.

### Immunofluorescence and TUNEL staining

Cells were fixed in 4% paraformaldehyde for 15 min and washed twice with PBS-T. After permeabilization with PBS containing 0.1% Triton-X, cells were washed twice with PBS-T, blocked with PBS containing 3% BSA for 1 hour at room temperature, and incubated with primary antibody (Santa Cruz, 1∶200) overnight at 4°C. Then cells were washed three times with PBS-T, incubated with Alexa flour 488 conjugated secondary antibody (1∶500) and DAPI (1∶500) for 2 hours at room temperature. Unbound secondary antibody was removed by three washes with PBS-T. Fluorescent images were captured using a fluorescent microscope (IX70, Olympus) equipped with CCD camera (RT Color, Diagnostic, Spot Software V4.0.9).

To identify apoptotic cells, TUNEL staining was performed using the *in situ* cell apoptosis detection kit (Roche) according to the manufacturer's instructions. Briefly, cells were fixed with 4% paraformaldehyde for 30 min. Terminal deoxynucleotidyl transferase (TdT) and biotin-11-dUTP reactions were performed for 1 hour at 37°C. Fluorescent images were captured using a fluorescent microscope equipped with CCD camera (above).

### Histochemistry

Calcification was identified with Alizarin Red S staining, as described previously [13]. To measure calcium accumulation during differentiation, cultures were digested with 100 µl of with 10% formic acid. Values were calculated using the Arsenazo III (DCL), which was measured along with the samples at 650 nm in a Benchmark Plus microplate spectrophotometer (BioRad). The readings were then normalized to dsDNA content which was measured using a Bio Photometer (Eppendorf).

Lipid accumulation was identified with Oil Red O staining at 10 days of differentiation, as described previously [17]. Nuclei were stained by hematoxylin. Intracellular triglyceride accumulation was measured using AdipoRed (Lonza), with excitation 485 nm and emission 572 nm in a SpectraMax M2 (Molecular Devices). Readings were normalized to dsDNA content which was measured using a Bio Photometer.

### Scanning electron microscopy

After the fixation, aggregates were coated with gold using a Hummer 1 Sputter Coater. The samples were examined with by SEM (Phillips/FEI ESEM XL-30) operated at working distance of 10.7 mm and an accelerating voltage of 20 kV.

### *In vitro* and *In vivo* implant assay

Aggregates cultured in static and stirred suspension were dissected at 15 and 30 days. Specimens were fixed in 4% paraformaldehyde. After dehydration in ascending concentrations of ethanol and xylene, the specimens were embedded in paraffin. The paraffin sections were then deparaffinized, hydrated, and stained with hematoxylin and eosin (H&E) and Alizarin Red S (Wako).

In order to determine the osteogenic potential of cultured cells to differentiate *in vivo*, 15 day aggregates were injected into the muscle of SCID mice, using a 26 gauge syringe. After 4 weeks, animals were sacrificed and tissues at the point of injection were dissected. Specimens were fixed in 4% paraformaldehyde. After dehydration in ascending concentrations of ethanol and xylene, the specimens were embedded in paraffin. The paraffin sections were then deparaffinized, hydrated, and stained with H&E, Toluidine Blue, and Methylene Blue according to standard procedures. Methylene Blue consisted of methylene blue (Sigma) and 0.03% basic fuchsin (Sigma).

### Statistical analysis

Means ± S.E.M. were calculated and statistically significant differences between two groups were determined using the Student's *t*-test at *P*<0.05 (Excel, Microsoft).

## Results

### ESC osteoblast differentiation in micro-mass culture

Previously, we differentiated ESCs towards the chondrocyte lineage using micro-mass culture [16]. In response to differentiating agents, many cells underwent apoptosis, while others left the micro-mass, forming small cartilaginous aggregates in suspension. In this study, we attempted use a similar micro-mass approach in order to differentiate ESCs towards the osteoblast lineage. Here, we also compared serum-containing and -free conditions. We also compared dexamethasone (Dex) and vitamin D3 (VD3), which have both previously been used as osteo-inducers in a background of ascorbic acid (AA) and β-glycerophosphate (β-GP) [3]–[7].

**Figure 1:**
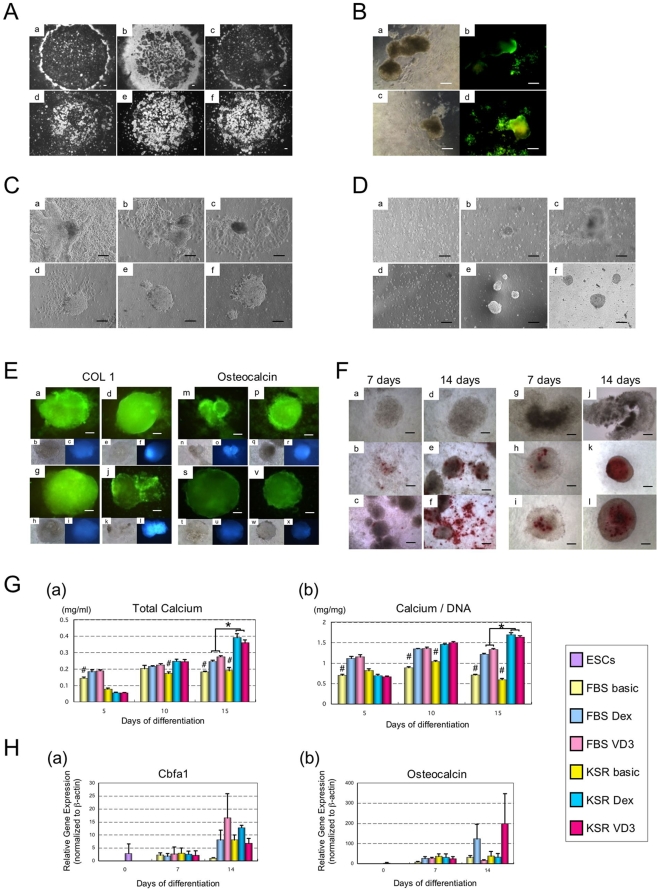
Micro-mass directed ESC differentiation towards the osteoblast lineage. (A) Morphological changes on day 4 of differentiation between the six media conditions tested: FBS-basic (a), FBS-Dex (b), FBS-VD3 (c), KSR-basic (d), KSR-Dex (e), KSR-VD3 (f). Scale bars represent 100 µm. (B) Apoptotic cells were observed using TUNEL on day 4 of differentiation in KSR-Dex (b) and KSR-VD3 (d). The corresponding brightfield channels demonstrates cell position (a and c respectively); scale bars represent 100 µm. (C) Re-attached aggregates on day 7 show significant differences at day 7 between the six media types: FBS-basic (a), FBS-Dex (b), FBS-VD3 (c), KSR-basic (d), KSR-Dex (e), KSR-VD3 (f). (D) After trypsinization on day 7 of differentiation, aggregates also show differences between the six media types: FBS-basic (a), FBS-Dex (b), FBS-VD3 (c), KSR-basic (d), KSR-Dex (e), KSR-VD3 (f); scale bars represent 100 µm. (E) Expression of osteoblast-related proteins, COL 1 and osteocalcin using immunofluorescence on day 7 of differentiation: FBS-Dex (a, m), FBS-VD3 (d, p), KSR-Dex (g, s) and KSR-VD3 (j, v). Brightfield (b, e, h, k, n, g, t, w) and DAPI (c, f, i, l, o, r, u, x) are also shown; scale bars represent 50 µm. (F) Using Alizarin Red S staining, calcification with red appearance was observed in culture dish on day 7 and 14 of differentiation: FBS-basic (a, d), FBS-Dex (b, e), FBS-VD3 (c, f), KSR-basic (g, j), KSR-Dex (h, k), KSR-VD3 (i, l); scale bars represent 50 µm. (G) Calcium content per micro-mass spot (a) and normalized against DNA (b) was determined; data is expressed as mean ± SD (n = 3) per well. ^#^ represents a significant difference between FBS-Dex and VD3 or KSR-Dex and VD3; P<0.05 with Student's *t-*test. * represents a significant difference between FBS and KSR, P<0.05 with Student's *t-*test. (H) Expression of osteoblast-related mRNAs (Cbfa1, osteocalcin) was analyzed using real-time PCR at 0 (ESCs), 7 and 14 days. Data is expressed as means ± SD (n = 3) per lane.

To examine cell growth and differentiation, we tested six media formulations: FBS-basic, FBS-Dex, FBS-VD3, KSR-basic, KSR-Dex and KSR-VD3. In the FBS treatment groups, cells expanded peripherally to the central micro-mass (Fig. 1Aa, b, c). In the KSR treatment groups, cells aggregated spontaneously and lifted off the culture surface after 4 days (Fig. 1Ad, e, f). Similar to our previous chondrogenesis study, apoptosis aggressively occurred upon the initial addition of differentiation factors (Fig. 1B); many cells left the micro-mass, formed aggregates in suspension and then reattached to the plates [16].

Aggregates that formed displayed striking morphological differences between the FBS and KSR treatment groups. KSR-treated aggregates retained a uniform and compact formation compared to FBS-treated aggregates, which displayed irregular and undefined outgrowths (Fig. 1C). Interestingly, aggregates that formed in the KSR-Dex and KSR-VD3 treatments groups could not be dissociated with trypsin (Fig. 1D), while those formed under all other conditions could.

In the FBS-Dex, FBS-VD3, KSR-Dex and KSR-VD3 treatments groups, expression of the osteoblast related proteins COL1 and osteocalcin, was observed by 7 days (Fig. 1E). Calcification, as indicated by Alizarin Red S, staining was observed at 7 days and intensified by 14 days (Fig. 1F). As well, total calcium content was enhanced during differentiation but was especially high in the KSR-Dex and KSR-VD3 treatments groups. However, no significant difference was observed when comparing Dex and VD3 inducing conditions (Fig. 1G). Using real-time RT-PCR, we also examined mRNA levels for the osteoblast markers Cbfa1 and osteocalcin. Although Cbfa1 and osteocalcin were expressed in differentiated cells, these genes were not up-regulated dramatically compared to FBS-basic and KSR-basic conditions (Fig. 1F). As we did not observe a similar phenomenon in our previous chondrogenic differentiation experiments, this result caused us suspect that something in the culture methodology was disturbing osteoblast lineage commitment during ESC differentiation.

### Adipogenesis interferes with osteoblast differentiation in adhesion culture

**Figure 2:**
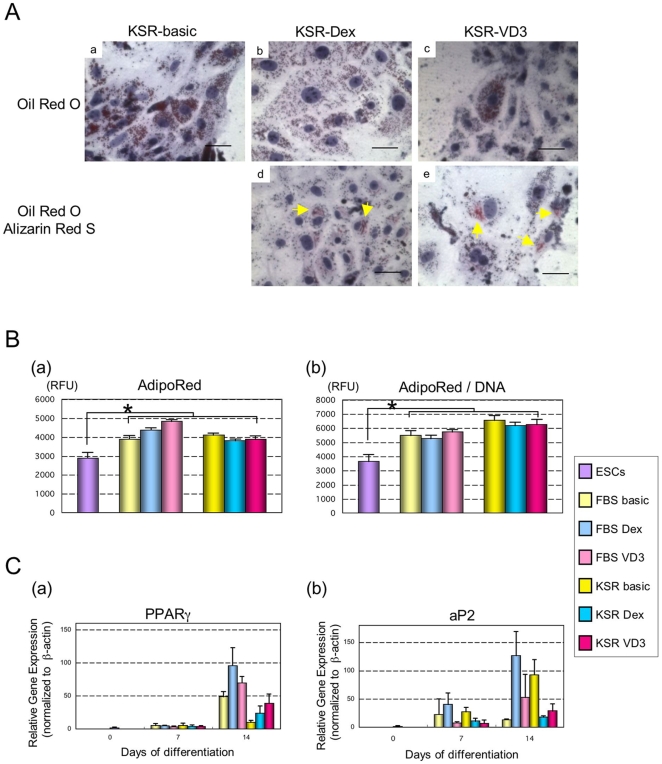
Adipogenesis during ESC osteoblast differentiation in static culture. (A) Using Oil Red O staining, lipids with dark red appearance were observed in re-attached cells on day 10 of differentiation in static: KSR-basic (a), KSR-Dex (b), KSR-VD3 (c). Oil Red O co-staining with Alizarin Red S shows both lipid and calcification within re-attached cells in KSR-Dex (d) and KSR-VD3 (e); scale bars represent 20 µm. (B) Lipid accumulation per micro-mass spot was determined on day 10 of differentiation (a). Lipid accumulation was also normalized against DNA content to determine lipid synthesis per cell (b); data is expressed as mean ± SD (n = 3) per well. * represents a significant difference between undifferentiated and differentiated ESCs; P<0.05 with Student's *t-*test. (C) The expression of adipocyte-related mRNAs (PPARγ, aP2) was analyzed using real-time RT-PCR at 0 (ESCs), 7 and 14 days; data is expressed as means ± SD (n = 3) per point.

As osteoblasts and adipocytes share a common progenitor, we investigated whether adipogenesis might be a disturbing factor that prevented efficient osteoblast differentiation. Adipocytes accumulates lipid within their cells during development. Using Oil Red O staining, we observed significant lipid accumulation in cells by day 10 of osteogenic differentiation KSR-Dex, KSR-VD3, compared to KSR-basic medium (Fig. 2A). Interestingly, when co-stained with Oil Red O and Alizarin Red S, many of the lipid containing cells also showed signs of mineralization. This lipid accumulation was confirmed biochemically using AdipoRed, where we observed a considerable increase in lipid accumulation in osteogenic conditions compared to undifferentiated controls (Fig. 2B). When we examined the expression of adipocyte-related genes using real-time RT-PCR, we observed that both adipocyte markers, PPARγ and aP2, were significantly up-regulated in osteoblast differentiations compared to undifferentiated controls (Fig. 2C).

### ESC Osteogenesis in static suspension culture

**Figure 3:**
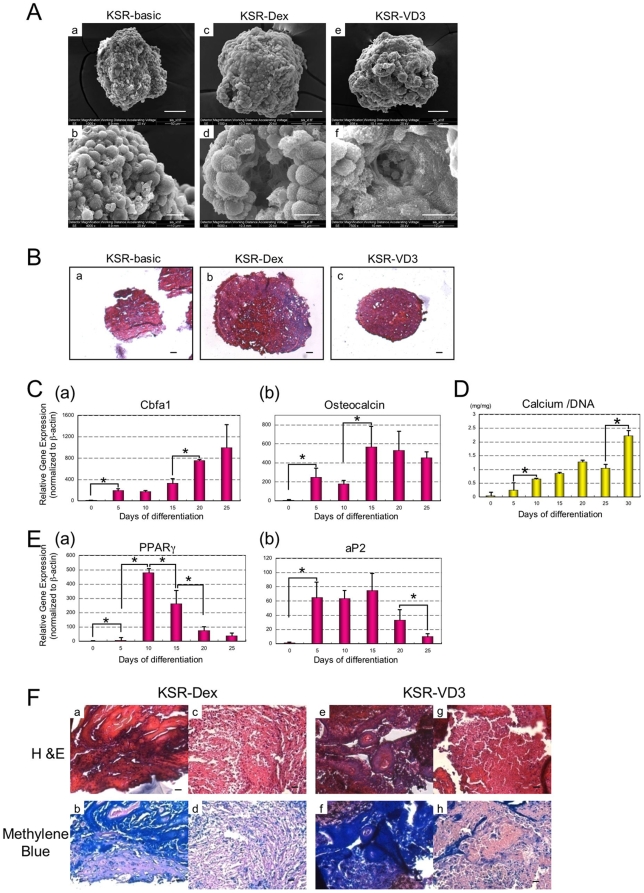
Bone-like tissue formation *in vitro* and *in vivo*. (A) The ultra-structure of aggregates was analyzed by SEM on day 15 of stirred suspension culture: KSR-basic (a, d), KSR-Dex (b, e), KSR-VD3 (c, f). Low magnification (a, b, c), scale bars represent 50 µm; high magnification (d, e, f), scale bars represent 10 µm. (B) Histological sections of aggregates were analyzed by hematoxylin and eosin (H&E) staining after 15 days of differentiation: KSR-basic (a), KSR-Dex (b), KSR-VD3 (c); scale bars represent 20 µm. (C) Expression of osteoblast-related mRNAs (Cbfa1, osteocalcin) was analyzed using real-time PCR during ESC differentiation in KSR-VD3 static suspension culture. (D) Calcium content was analyzed during ESC differentiation in KSR-VD3 static suspension culture and normalized against DNA content. (E) Expression of adipocyte-related mRNAs (PPARγ, aP2) was analyzed using real-time RT-PCR during ESC differentiation in KSR-VD3 static suspension culture; data is expressed as means ± SD (n = 3) per lane. * represents a significant difference between two conditions tested; P<0.05 with Student's *t-*test. (F) Following 15 days of differentiation in static suspension culture, bone (a, b, e, f) and epithelium (c, d, g, h)-like tissues were observed *in vivo* upon transplantation into SCID mice: KSR-Dex (a, b, c, d) and KSR-VD3 (e, f, g, h); H&E (a, c, e, g) and Methylene Blue (b, d, f, h) staining, scale bars represent 20 µm.

It has previously been reported that plating cell density regulates a lineage commitment switch between osteogenic and adipogenic differentiation [14], [18]. Reconciling that cell density, might be high within reattached aggregates, we investigated whether osteogenic differentiation might be improved in suspension culture. When differentiated in serum free conditions, both KSR-Dex and KSR-VD3, aggregates formed spontaneously within micro-drops (Fig. 1A, B). Further, serum-free culture also prevented aggregates from reattaching to dishes. These aggregates were hence collected and cultured in static suspension. By 15 days in suspension culture, these aggregates consisted of round cells, which formed a spherical structure. Interestingly, in both KSR-Dex and KSR-VD3 conditions, these spherical aggregates displayed a porous-like tissue bearing a collagen-like fiber network, which connected cells (Fig. 3A, B).

We examined mRNA levels for osteoblast and adipocyte genes during differentiation in KSR-VD3 using real-time RT-PCR. The osteoblast transcription factor Cbfa1 was up-regulated during differentiation, while expression of osteocalcin was up-regulated and stable from 15 days onwards (Fig. 3C). Similarly, calcium content also became up-regulated during differentiation (Fig. 3D). On the other hand, expression of the adipocyte related transcription factor, PPARγ, peaked at 10 days and decreased throughout the remainder of the differentiation period. Similarly, expression of adipocyte marker, aP2, was diminished from day 15 onwards (Fig. 3E). These results suggested that in static suspension culture, osteogenesis became enhanced, while adipogenesis was suppressed.

Epithelial-mesenchymal interactions play a key role in intermembranous ossification resulting in the development of flat bones and fusion with epithelium [14]. After transplantation, aggregates formed both bone and epithelium-like tissue *in vivo* (Fig. 3F). Cartilage and adipose tissue were not observed. These results indicated that ESCs, which were differentiated directly towards the osteoblast lineage in static suspension culture may have formed bone though an intermembranous ossification without adipocyte or chondrocyte involvement.

### Cells remain pluripotent in stirred suspension culture differentiations

**Figure 4:**
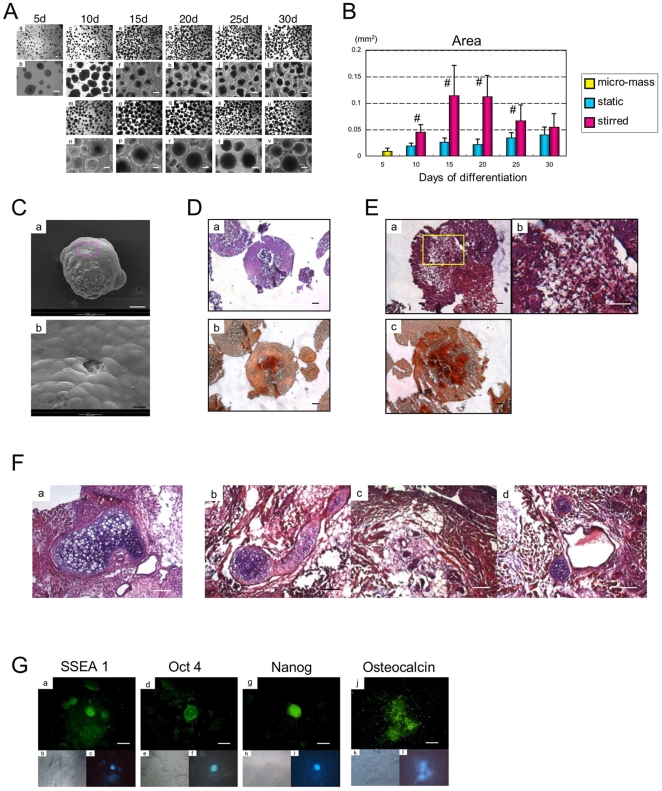
Trapped, undifferentiated and differentiated ESCs within aggregates in stirred suspension culture. (A) ESC-derived aggregates displayed dramatic changes in morphology in static and stirred suspension culture using KSR-VD3 medium: micro-mass (a, b), static suspension (c-l), stirred suspension (m-v); scale bars represent 100 µm. (B) The average area of aggregates in a static or stirred suspension culture was determined during culture; data is expressed as means ± SD (n = 10) per lane. ^#^ represents a significant difference between static and stirred suspension; P<0.05 with Student's *t-*test. (C) The ultra-structure of day 15 stirred suspension aggregates was analyzed by SEM: Low magnification (a), scale bars represent 100 µm; High magnification (b), scale bars represent 10 µm. (D, E) Sections of day 15 (D) and day 30 (E) stirred suspension aggregates were analyzed by H&E (a) and Alizarin Red S (b, c); scale bars represent 20 µm. (F) When transplanted into SCID mice after 15 days of *in vitro* differentiation, teratomas including cartilage and adipose tissues were observed by H&E staining *in vivo* (a). After 30 days of *in vitro* differentiation, resulting teratomas included cartilage (b), adipose (c), blood and blood vessel (d) were observed. Scale bars represent 50 µm. (G) Expression of pluripotency markers: SSEA1 (a), Oct4 (d) and Nanog (g) and osteoblast-related protein osteocalcin (j) was analyzed by immunofluorescence after aggregates were allowed to outgrow onto plates; Brightfield (b, e, h, k); DAPI (c, f, i, l); Scale bars represent 50 µm.

We next investigated aggregate differentiation in stirred suspension culture. Here, the size of aggregates increased dramatically compared to static suspension culture (Fig. 4A, B). This size peaked at 20 days and then gradually decreased over time (Fig. 4B). When we used scanning electron microscopy to examine the morphology of aggregates following 15 days of differentiation, we observed them to be radically different than similar aggregates maintained by static suspension culture. Rather than having well defined cell outlines, and channels, aggregates displayed a smoothened surface, where borders between cells were filled significantly with ECM (Fig. 4C). Upon histological sectioning, we observed two predominant cell types, by 15 days and 30 days of differentiation, mineralized cells in the inner core surrounded by non-mineralized cells, lying underneath a layer of extracellular matrix (ECM) (Fig. 4D, E).

Upon transplantation into SCID mice, both day 15 and day 30 differentiated aggregates formed teratomas (Fig. 4H). These results suggested that a proportion of cells remained pluripotent within these aggregates after 30 days of differentiation in the absence of leukemia inhibitory factor (LIF). When these aggregates were outgrown onto culture dishes and maintained without LIF, ESC-like colonies were appeared, which expressed the ESC markers SSEA1, Oct4 and Nanog (Fig. 4G). The same outgrowths also showed osteocalcin positive cells (Fig. 4Gj–l).

## Discussion

The application of ESCs in bone regenerative medicine requires the development of efficient of methodologies for differentiating ESCs into the osteoblast lineage *in vitro* and functional bone formation *in vivo*. In this study, we developed an effective approach to *in vivo* bone formation based on the process of intermembranous ossification. Our study has revealed some key factors that are necessary for efficient bone regeneration.

Previously, we demonstrated the ability to differentiate ESCs into chondrocytes using a micro-mass approach, without the need for an embryoid body (EB) step [16]. ESCs were affixed to the bottom of micro-drop cultures and treated with differentiation factors. In response, many cells underwent apoptosis, while others left the micro-mass, forming small cartilaginous aggregates in suspension. As a follow on study, we sought to investigate whether a similar process could be used to differentiate ESCs into osteoblasts. Much like our previous study, discordant cells underwent apoptosis (Fig. 1B), while others left the micro-mass and formed small aggregates (Fig. 1C, D). These aggregates were subsequently used to explore differentiation in static and stirred suspension culture.

When aggregates were outgrown onto tissue culture plates, they continued to show the hallmarks of osteoblast differentiation (Fig. 1E–G). However, although osteoblast-related genes were expressed, these genes were not up-regulated dramatically compared with basic medium (Fig. 1H). Moreover, unlike our previous cartilage study, which resulted in cartilage formation upon transplantation, these aggregates did not form bone *in vivo* (data not shown).

Cell density has previously been shown to limit the efficiency of mesenchymal stem cell (MSC) osteoblast differentiation in favor of adipocyte differentiation [18]. Indeed, when we examined these aggregate outgrowths for signs of adipogenesis, we observed a considerable amount of Oil Red O staining within the outgrowth. In some cases, cells displayed both lipid accumulation and mineralization, suggesting that cells were between states of commitment. Indeed, the osteoblast and adipocyte lineages are closely related [9], [10]. Previously, we have demonstrated that the balance between osteoblast and adipocyte commitment can be controlled through the expression levels of a single transcription factor PPARγ [13]. In our studies, we observed both PPARγ and the adipocyte marker aP2 were highly regulated in adhesion cultures, suggesting that adhesion cultures positively influenced adipogenesis while suppressing osteogenesis.

In sharp contrast, this adipogenic interference did not occur when aggregates were cultured in suspension. In static suspension, cells expanded considerably in size, pushing the bounding envelope outwards. During differentiation in static suspension culture, the osteoblast transcription factor Cbfa1 was up-regulated, whereas the adipocyte transcription factor PPARγ became down-regulated from 15 days onward. When transplanted into animals, these aggregates also formed bone without cartilage and adipose tissue (Fig. 3F).

While, the adipogenic interference effect that we observe may be related to cell density, we should not ignore the fact that the expression of cellular adhesion proteins may be different in cells forming aggregates compared to cells adherent to substrates. For example, we have previously observed that ESC aggregates are very reliant upon E- and N-cadherin, whereas ESC adhesion cultures are reliant on focal adhesion proteins such as vinculin (unpublished results). Indeed, the expression of adhesion proteins has previously been found to regulate stem cell differentiation [19].

At 15 days, cells in aggregates became connected with collagen-like fibre network and formed a porous tissue (Fig. 3A, B). These aggregates also formed bone without any evidence of adipose tissue *in vivo* (Fig. 3F). Additionally, we observed no cartilage at the transplantation site suggesting that bone formation occurred via the process of intermembranous ossification. During development, intermembranous ossification, normally involves bone mineralization upon a membranous, connective tissue anlagen [18]. In this study, both bone and epithelial-like tissues were found at the site of transplantation (Fig. 3F). Here, it is likely that epithelial-mesenchymal interactions were involved in the commitment of progenitor cells to the osteogenic fate.

Static suspension culture is mass transport limited resulting in reduced cell growth and hypoxia [20]. To overcome such limitations, stirred suspension culture was also explored for osteoblast differentiation. The use of a stirred suspension culture produces shear stress on cell aggregates, while providing adequate mass transport and oxygenation and supporting 3D tissue-like growth. Although stirred suspension culture resulted in a significant growth of aggregates (until about day 20), these aggregates changed considerably in cell morphology (Fig. 4A–C). Rather than being porous in nature, aggregates were intact bearing a smoothened surface. Histological analysis revealed an inner core of mineralized cells surrounded by a layer of non-mineralized cells lying underneath a layer of ECM (Fig. 4D, E).

Rather than form bone, these aggregates gave rise to teratomas when transplanted into animals. Recently, Taiani *et al*. has reported that ESC aggregates, which are differentiated directly towards the osteoblast and chondrocyte lineage in suspension bioreactors, also form tumors at high frequency and demonstrated that differentiated aggregates are rich in pluripotent cells based upon marker expression [21]. Unlike static culture differentiations, where pluripotency markers are down-regulated within one week of differentiation, this group demonstrated that Oct4 is highly expressed when cell are differentiated in suspension. When we allowed day 30 stirred suspension culture aggregates to outgrowth onto dishes, we observed ESC-like colonies, which expressed the pluripotency markers SSEA1, Oct4 and Nanog (Fig. 4G). Interestingly, these same outgrowths also displayed osteocalcin positive cells (Fig. 4Gj–l), suggesting that osteoblasts were also present in these tumorigenic aggregates.

Taiani *et al*., has suggested that the hydrostatic forces that are experienced by the aggregates may be the cause of pluripotency maintenance within aggregates cultured by stirred suspension [21]. It has also been reported that stimulated microgravity suppresses osteoblast differentiation [22], [23]. Indeed, it is interesting that within our aggregates, we observe what appears to be a legacy of differentiation, with a mineralized core in the centre of the aggregate, representing the original aggregates which formed in static suspension culture and a non mineralized cortex. These results suggest that in addition to the maintenance of pluripotency, stirred suspension culture may have also suppressed osteoblast differentiation.

In this study, we demonstrated that culture microenvironment significantly influences cell fate decisions in the osteoblastic differentiation of ESCs. ESCs that are primed to become osteoblasts using micro-mass differentiation can efficiently form osteoblasts if cultured as aggregates in static suspension culture. However, if resulting cells are forced into adhesion culture, their fate quickly changes in favor of the adipocyte lineage. Similarly, if exposed to hydrostatic forces associated with stirred suspension culture, osteoblast differentiation is suppressed in favor of the expansion of progenitor cells. Our results confirm that cell culture methods have a significant influence upon ESC differentiation and that care must be taken to optimize differentiation culture methodology before contemplating clinical applications.
